# Life Course Dynamics in the Health of Mothers Raising Children with Serious Conditions

**DOI:** 10.1177/00221465251353536

**Published:** 2025-08-03

**Authors:** Xuewen Yan, Robert Crosnoe

**Affiliations:** 1 University of Texas at Austin, Austin, TX, USA

**Keywords:** children with disabilities, life course, mothering, multiple children, parental health

## Abstract

Although raising children with serious conditions is known to be associated with poorer parental well-being, recent research following a life course perspective highlights how these associations accumulate over time. Expanding this perspective on long-term dynamics of this parental experience, this study examined how three conceptualizations of the “intensity” of this parental role—caregiving duration, cumulative transitions into this role, and the number of affected children—shaped maternal physical health in midlife. Fixed-effects modeling of panel data from the National Longitudinal Survey of Youth 1979 (n = 8,305) revealed that all three dimensions significantly predicted poorer maternal physical health, with particular salience for cumulative transitions and the number of affected children. These associations were generally weaker when mothers had higher income or greater labor force participation, although such buffering effects applied more consistently to labor force participation and specifically to repeated transitions and mothers of two (vs. one) affected children.

Parenthood is not only a transformative event featuring a mix of demands and rewards. It also is a key vector of health that can either undermine or promote well-being depending on the childrearing circumstances and the availability and accessibility of resources (Umberson, Pudrovska, and Reczek 2010). Parents of children with serious conditions—defined as physical, developmental, or emotional disabilities or chronic illnesses that require special care—face unique challenges in maintaining their own health (Ha et al. 2008). Such parenting can demand more intensive investment and engagement, often extending into the child’s adult life (Cha and Crosnoe 2022). This burden falls especially on mothers who, due to the persistent gendered division of domestic labor, shoulder most childrearing responsibilities (Calarco 2024). Thus, despite the many positive and fulfilling aspects of raising a child with a serious condition, there is an average tendency for mothers with this experience to report poorer physical and mental health, such as disrupted sleep, accelerated signs of aging, and greater risk of cardiovascular disease and mortality (Barker et al. 2012; Cohn et al. 2020; Ha et al. 2008; Meltzer and Mindell 2006; Stainton and Besser 1998).

This significant, policy-relevant line of research can be enriched by a life course perspective, which encourages the contextualization of the linked lives between parents and children within long-term temporal dynamics and systems of social stratification (Elder, Johnson, and Crosnoe 2003). This perspective calls for a fuller accounting of the parenting “career” amid inequalities in resources and challenges. For example, Cha and Crosnoe (2022) used a life course perspective to extend the often studied case of young mothers of young children with serious conditions to the underexamined case of aging mothers of adult children, with special attention to maternal education. This longer-term lens identified how duration in this maternal status played a critical role in women’s physical (but not mental) health, specifically, how this status might lead to physical wear and tear over time that mattered more than recent or even contemporaneous experiences of such mothering. This life course pattern aligns with the concept of cumulative disadvantage and related models contrasting “dosage” and “threshold” effects of some social, structural, or policy factor (Dannefer 2003; Fuller et al. 2017; Galster 2012).

Following a life course perspective, therefore, this study unpacked this phenomenon and contextualized it within broader dimensions of socioeconomic stratification in the United States using decades of data from the National Longitudinal Survey of Youth: 1979 (NLSY79). It did so by comparing three conceptualizations of “dosage,” in other words, how much women were “exposed” to the experience of being a mother of a child with a serious condition and the consequent implications for their physical health (to avoid medicalizing this maternal status, we used the term “intensity” rather than “dosage” or “exposure”). These conceptualizations included the duration of raising children with serious conditions, the accumulation of transitions into this role, and number of children with serious conditions whom mothers raised. Our first research question addressed which conceptualization mattered most to understanding maternal health disparities at midlife. Our second question addressed whether the relative comparison of these conceptualizations in the health of mothers at midlife depend on fluctuations in their incomes and labor force participation, two key markers of positions given the socioeconomic system of the United States. We explored these questions by tracing the life course histories of women in the NLSY79 according to whether and when they raised children with serious conditions since they first became mothers and connecting these histories to their physical health at ages 40, 50, and 60 with fixed-effects modeling. Such research makes several contributions, including using life course concepts to identify critical groups and windows of intervention, linking family health to broader forces of socioeconomic inequality that illuminate the heterogeneity of parenting, and employing methods to improve causal inference in a literature that is susceptible to endogeneity biases.

## Background

### Children’s Serious Conditions and Mothers’ Health

Because medical advances increase the survival rates and life expectancy of children with serious medical complexities, the number of parents engaged in short- and long-term primary care for such children has grown (Hatzmann et al. 2008). Following the persistence of gendered caretaking and parenting, most of these parents are women (Calarco 2024). There is a general sense that this mothering experience can undermine their own health as they face financial insecurities due to incomplete medical insurance coverage and compromised employment, everyday stigma associated with a child who may not be typically developing, existential worries about the child’s futures (including future care), role conflicts between caregiving and other personal and familial pursuits, and the likelihood of continued parenting involvement into a child’s adult life when other parents are “done” (Green 2007; Ha et al. 2008; Hastings 2002; King et al. 2006; Østerud, Skjønsberg, and Albertini Früh 2024; Raina et al. 2005). Several meta-analyses and reviews covering a range of national contexts document this link between raising a child with a serious condition and maternal well-being (Cohn et al. 2020; Cousino and Hazen 2013). There are, of course, many such mothers doing fine, and the reality is that these mothering experiences—like all mothering experiences—can bring great rewards that counterbalance such strains and stressors (King et al. 2006). Yet, on average, raising a child with a serious condition is, at least statistically speaking, a risk factor.

Coupling life course perspectives with longitudinal designs, however, has revealed many ways that this potential risk is dynamic. As mothers age and their children with serious conditions develop, their relationships, needs, circumstances, and experiences can evolve. That evolution can encompass changes in children’s conditions, mothers’ gradual adaptation to the role so that they can better enjoy its rewards, and/or an amplification of the toll of challenges and strains as they persist (Cha and Crosnoe 2022). What matters, then, is the long arc of the parenting career and what it means for both mother and child. As already noted, some evidence from studies using a life course perspective points to the possibility that just like the rewards of this particular maternal status may be overcome by its strains at any one point, the cumulative toll of this experience over longer periods of time may undercut the potential for adaptation. In other words, aging mothers in this maternal role tend to have poorer health than other mothers, even those in similar childrearing circumstances, the longer they have been raising children with serious conditions (Cha and Crosnoe 2022).

This pattern represents a potential dosage effect, or the phenomenon in which the association between some factor (e.g., a condition or an intervention) and outcome strengthens as the amount of that factor increases (Burchinal et al. 2016). The general goal of this study was to consider the different forms that such a dosage effect (again, referred to as “intensity”) could take. Drawing on work about family structure and process and exploiting data that improve on more simplistic treatments of family life in past research (e.g., short time windows, nonrepeated measurement, data on only one child), we focus on three here.

First, “duration” refers to the span of time rearing a child with serious conditions. Following the cumulative stress model (Hoyert and Seltzer 1992), duration should matter through the buildup of strains that wear down mothers over time when such parenting requires more and prolonged physical exertion, overactivates the body’s stress response system, and accelerates typical aging processes, such as oxidative stress and the erosion of telomere length (Epel et al. 2004). As already noted, the 2022 study by Cha and Crosnoe documented the explanatory power of this conceptualization, although without comparing it to other conceptualizations. At the same time, some research has revealed evidence for a competing model of adaptation in that associations between children’s disabilities and poorer parental well-being were weaker for older parents than younger parents (Ha et al. 2008).

Second, the “accumulation of transitions” refers to the number of times mothers assume this maternal role, anew or again, across their parenting careers. Many serious conditions are neither permanent nor continuous. Instead, a child could move through a cycle of diagnosis, temporary remissions, recoveries, and relapses, and another child can recover from one serious condition and then develop a second condition years later. Such transitions take on meaning because of ample evidence from other domains about the disruptive nature of repeated social and personal transitions and any associated instability they trigger. For example, multiple changes in family structure are negatively associated with adolescents’ well-being (Cavanagh 2008), family income volatility appears to undermine longer-term educational attainment (Hardy 2014), and people (especially women) report greater stress when they experience more shifts in social roles and settings over the course of a day (Cornwell 2013).

Third, “number of children” refers to the possibility that there may be multiple children in a family with serious conditions. Many studies focus on a single child for practical reasons, such as patient-oriented data collection in medical research (Meltzer and Mindell 2006). There also may be limited resources to collect or analyze data from multiple children in population studies, leading to the selection of a focal child through purposeful (e.g., the oldest or first diagnosed) or more random sampling (Cha and Crosnoe 2022; Ha et al. 2008). Yet any mother–child dyad is embedded in and influenced by the full family system, including siblings (Cox and Paley 1997). Many serious conditions also arise out of shared genetic and environmental underpinnings so that the presence of one child with a serious condition raises the likelihood of more (Hemminki and Li 2004). If children with serious conditions tend to cluster in families, then the consideration of intensity as cumulative strain would suggest that having more such children increases the magnitude of caregiving in ways that will further undermine maternal health (Burchinal et al. 2016). An alternative perspective from birth order research suggests a resource dilution hypothesis—that the health risks of raising children with serious conditions may be concentrated around the first diagnosed child, with additional children who are diagnosed only marginally increasing this risk (Barclay, Lyngstad, and Conley 2021; Buckles and Kolka 2014).

### Socioeconomic Variability in Dynamic Links between Mothering and Maternal Health

A life course perspective motivates the dynamic treatment of the linked lives of mothers and children with serious conditions through the varied temporal conceptualizations of intensity, but it also advocates for contextualizing these dynamic linked lives within more proximate ecologies of everyday life and the broader systems of society. We do so here by considering the socioeconomic circumstances of women. Our argument is that both income and labor force participation reflect women’s positions in the socioeconomic hierarchy in which these three conceptualizations of intensity might be less related to their health; in other words, mothers in such positions might be better equipped to weather the strains of this particular maternal status and realize its rewards to maintain better health over longer periods of time.

Why might income play such a buffering role, disrupting the association between greater intensity of raising a child with a serious condition and poorer maternal health? Income drives socioeconomic stratification and determines access to resources to cope with challenging situations (Phelan, Link, and Tehranifar 2010). Mothers with higher family incomes have more purchasing power to secure higher quality health care, stable childcare help, high-cost medical equipment to assist with children’s functioning, and enrichment activities that foster social connections and health literacy. Higher incomes also bring various essential benefits, such as a sense of financial security for everyday routines, a nutritious diet, and easier transportation, all of which could make a parent less susceptible to the stress and strain associated with caregiving. Following conceptual models positing that health-promoting resources can matter more in the face of health risks (e.g., resource substitution; Ross and Mirowsky 2006), mothers raising children with serious conditions may gain more from additional income and what it can buy than otherwise similar mothers whose children do not have such conditions.

Why might labor force participation also play such a buffering role? Paid work is a documented social determinant of health that protects and enhances well-being throughout the life course, including and especially for women (Caputo, Pavalko, and Hardy 2020; Frech and Damaske 2012). Despite persistent social concerns (including among past scholars) about women’s work–family conflicts, those employed for pay tend to have better health than other women, even when accounting for their selection into employment (Klumb and Lampert 2004; Repetti, Matthews, and Waldron 1989). Although income contributes to this pattern, it cannot fully explain it. Women who transition into paid employment experience improved well-being after controlling for job characteristics and income levels (Lu et al. 2023). There are intricate social and psychological mechanisms at play in such patterns, including the potential to find information and support networks, greater efficacy, social status, outlets for achievement and skill development, and a respite from family responsibilities and stressors in the workplace (Goffman [1963] 2009; Hochschild 2003; Mirowsky and Ross 2007). Again, if such factors promote health, they potentially do so more among women in circumstances that strain health—such as mothers raising children with serious conditions—than for other women in terms of both reducing such strains and allowing rewards to come through.

### Study Aims

Following this life course perspective, our first aim is to test the hypotheses that the dynamic links between raising a child with a serious condition and mothers’ physical health over many years should be sensitive to intensity. Specifically, they should get stronger with each additional year that a mother has this particular role, with each new transition into this role, and with each additional child with serious conditions the mother is raising. There are possible alternate hypotheses (e.g., adaption, dilution) that need to be considered, but these expectations are most in line with past research and theory and best capture the spirit of life course research on health. The second aim is to test the hypothesis that the role of intensity in health should be constrained by socioeconomic advantages. Specifically, the three forms of intensity should matter less to the health of mothers of children with serious conditions when they have higher incomes and are more active in the labor force.

## Data and Methods

### Data

The NLSY79 offers large-scale, long-term linkages between U.S. mothers and their children, allowing us to construct a longitudinal history of each child’s serious conditions while accounting for the personal and family contexts of the mothers. It is a nationally representative study of 12,686 U.S.-residing men and women who were ages 14 to 22 at their first interview in 1979. Respondents were followed annually until 1994 and then biennially over 27 rounds, with the most recent in 2020. In 1986, the NLSY79-Child and Young Adult data collection began, tracking all children born to the female respondents of the NLSY79. Beginning in 1998, as the oldest mothers in the cohort reached age 40, they were asked to complete an extended 40+ health module in addition to the regular NLSY79 follow-up. Subsequently, while the NLSY79 continued its regular biennial schedule, a similar 50+ health module was administered starting in 2008 and a 60+ module starting in 2018 alongside the NLSY79 interview. The 40+, 50+, and 60+ modules formed the basis of our health outcome measures and were completed when a respondent approaches or has reached the respective age threshold.

By the most recent wave in 2020, 4,943 mothers had been interviewed. We excluded mothers who did not participate in any of the 40+, 50+, or 60+ modules (n = 1,220) and those who had missing values on health measures (n = 11) or the focal predictor variables (n = 30). This analytical sample consisted of 8,305 observations nested within 3,682 mothers. The primary reason there were 8,305 observations instead of 11,046 (i.e., 3,682 women at three waves of data) was because over half of the mothers had not yet reached age 60 to complete the 60+ module by 2020. All observations were retained in the main analyses given the suitability of multilevel modeling techniques for unbalanced data (Snijders and Bosker 2011). We note that results remained substantively similar when analyses were restricted to the balanced sample or the sample of responses from the 40+ and 50+ modules.

### Measurement

#### Mother’s physical health

The 40+, 50+, and 60+ health modules included a physical composite score derived from the clinically validated and widely used Short-Form 12-question scale. This scale assessed both physical (e.g., mobility, physical pain, self-reported health) and mental (e.g., emotional distress, feelings of calmness) health. Physical composite scores (see Table 1) were calculated using the NLSY79’s proprietary scoring scheme and standardized to have a mean of 50 (the U.S. national adult average), a range of 0 to 100, and a standard deviation of 10. We also tested the mental composite score as an outcome but omitted those results here because, similar to Cha and Crosnoe (2022), we found no statistical association between measures of children’s illness histories and maternal mental health (see Appendix A in the online version of the article).

**Table 1.** Descriptive Statistics for the Study Sample (National Longitudinal Survey of Youth: 1979, *n* = 8,305).

Variable	*M*/Proportion	*SD*	Range
Physical health score	49.08	10.57	10.37–66.63
Raising child with serious condition			
Ever (ever serious)	40.26%		0–1
Duration (number of waves)	1.04	1.75	0–12
Cumulative transitions (number transitions)	.59	.88	0–6
Number of children with serious conditions	.55	.78	0–5
Number of children with serious conditions (categorical)			
0	59.74%		
1	28.89%		
2	8.86%		
3 or more	2.52%		
Socioeconomic circumstances			
Number of weeks working per year	35.64	15.07	0–52
Family income (IHS transformed)	10.73	1.25	0–13.50
Maternal covariates			
Number of children overall	2.45	1.20	0–11
Ever smoked	55.39%		0–1
Ever in poverty	51.34%		0–1
Years of education	13.25	2.47	0–20
Marital history			
Stably married	40.87%		
Never married	10.05%		
Disrupted	49.08%		
Survey age when health reported			
Age 40	42.79%		
Age 50	40.35%		
Age 60	16.86%		

*Note:* IHS = inverse hyperbolic sine.

#### Children’s serious conditions

At each wave, mothers—and children ages 15 or older—answered a series of questions about each child’s health conditions. We followed previous work to classify 14 medical conditions as serious: (1) disabilities (blindness, learning disability, speech impairments, orthopedic impairments), (2) chronic illness (asthma, cancer, diabetes, heart problem, respiratory disorder, anemia), (3) developmental disorders (autism, epilepsy, minimal brain dysfunction), and (4) emotional disorder (bipolar disorder; Cha and Crosnoe 2022; Van Cleave, Gortmaker, and Perrin 2010). For each time point, a child who had any of these conditions was categorized as having a serious condition.

Four variables captured the intensity of mothers’ experiences of parenting children with serious conditions. The first was a binary indicator of whether a mother had ever parented a child with serious conditions up to the corresponding year she reported her health (*ever serious*). Second, the *duration* variable counted the number of waves a mother reported having at least one child with a serious condition until she turned 40, 50, or 60, respectively. Third, *cumulative transitions* measured the number of transitions a mother experienced from having no child with serious conditions to having at least one such child from 1986 until she reached ages 40, 50, or 60. Finally, *number of children with serious conditions* counted the total number of children with serious conditions a mother parented by ages 40, 50, or 60. For comparison, we also provided a categorical version of this variable, including zero, one, two, or three or more children with serious conditions.

#### Covariates

Similar to the main predictors, all covariates were measured based on a summary of the respondent’s history of the designated variable between 1986 and the year the respondent turned 40, 50, or 60. *Labor force participation* was the average number of weeks a mother reported working per year. *Family income (IHS transformed)* was the inverse hyperbolic sine (IHS) transformation of the mother’s mean family income over this period. This transformation behaved similarly to the logarithm but had the advantage of directly accommodating zero values (Bellemare and Wichman 2020). We controlled for additional covariates that may have been simultaneously associated with maternal health and children’s illness history: the mother’s *parity* by ages 40, 50, and 60; her *years of education* at these time points; her *marital history*, categorized as stably married, never married, or ever disrupted (widowed, divorced, or separated); whether she *ever experienced poverty* during these years; and whether she *ever reported being a smoker* (measured as having smoked over 100 cigarettes in her lifetime). We also included dummy variables for the *age at survey* (ages 50 and 60 vs. 40) given the expected average decline in physical health during midlife. All the predictors considered were time-varying variables because we used fixed-effects modeling (see the following), which specifically exploited within-individual, between-time differences.

### Plan of Analyses

#### Fixed-effects modeling

Using fixed-effects regression models, we analyzed how changes in various dimensions of maternal exposure to children’s serious illness history across ages 40, 50, and 60 were associated with temporal changes in mother’s physical health. Fixed-effects models are well suited for panel analyses aimed at enhancing causal inference because they account for over-time clustering within the same individual and all sources of time-constant between-person heterogeneities by leveraging within-person variations over time. These models, therefore, did not estimate coefficients for time-invariant predictors, such as race or age at first birth, but yielded stringent estimates for the association between variations in health outcome and changes in a mother’s given characteristics across ages 40, 50, and 60. The fixed-effects estimators were generated by Equation 3, which resulted from subtracting a standard ordinary least squares Equation 1 for person i at time t from Equation 2, where both sides of Equation 1 were averaged across time:

(1) $$Y_{it}=\mu_{i}+\beta X_{it}+\gamma \nu_{i}+\alpha_{i}+\in_{it}.$$

(2) $$\bar{Y_{i}}=\mu_{i}+\beta {\bar{X}}_{i}+\gamma \nu_{i}+\alpha_{i}+\bar{\epsilon_{i}}.$$

(3) $$Y_{it}-{\bar{Y}}_{i}=\beta \left(X_{it}-{\bar{X}}_{i}\right)+\left(\epsilon_{it}-{\bar{\epsilon }}_{i}\right).$$

Thus, by subtracting the over-time averages from the time-varying attributes ( $X_{it}$ ), the fixed-effects models controlled for both observed and unobserved person-level attributes ( $\nu_{i}$ , $\alpha_{i}$ ) that were time-invariant or that varied at a constant rate over time while focusing on within-person temporal changes.

#### Internal moderator approach

Because of our focus on the “effects” of each conceptualization of maternal intensity (i.e., duration, repeated transitions, and the number of children with serious conditions) among mothers who had ever experienced rearing children with serious conditions, we fit models with these variables using an internal moderator approach (also known as the two-part predictor method). This approach employed interactions between a binary *ever serious* variable and the intensity variables to test the associations between the latter and the outcome within the subsample of mothers who ever had this experience. In the process, it yielded coefficients for other covariates from the full sample of mothers.

In the internal moderator method, a main effect for ever serious was included, but no main effects were fitted for the intensity variables because the intensity variables only had meaningful nonzero values when ever serious equaled 1. For example, for mothers with an ever serious value of 1, the coefficient for the interaction between ever serious and duration represented the effect of having a child with serious conditions for longer periods, conditional on ever having had this experience. Mathematically, this approach can be described as:

(4) $$\begin{array}{l} \mathrm{Health}\;=\mu_{0}+\beta_{1}Z+\beta_{2}Ever+\beta_{3}Ever\times \\ \;\;\;\;\;\;\;\;\;\;\;\;\;\;\;\;\;\;\;Dimension+\epsilon , \end{array}$$

where *Z* represented other covariates and *ever* was the ever serious variable that took a value of 0 if a mother never had a child with serious conditions by age 40, 50, or 60 and 1 if she had. Here, $\beta_{3}$ estimated the association of a specific dimension of maternal intensity and the health outcome when ever serious equaled 1. For mothers who had never had this experience, ever serious took a value of 0, meaning that their health outcome was predicted solely by the other covariates and the baseline effect of ever having the experience.

#### Model selection

The primary goal of this study was to compare the role in health of different dimensions of the intensity of women experiencing mothering children with serious conditions. Given the large number of possible model specifications (12 models), we first present model fit statistics from each specification to determine the best fitting model(s). We report the log likelihood (LL), the Akaike information criterion (AIC), and Bayesian information criterion (BIC) for each fitted model. A larger LL (e.g., a less negative value) represented a better model fit, and a smaller AIC or BIC indicated better fit. Among these metrics, we prioritized BIC because it penalized complex models with more predictors, thus facilitating the selection of the best predictive model while accounting for parsimony. We used AIC as a complementary criterion because it is particularly useful when the true model is highly complex or nonparametric (Chakrabarti and Ghosh 2011; Vrieze 2012). The six selected models were a baseline model with the ever serious predictor only, four models that each used one maternal intensity variable as a predictor (all applying the internal moderator approach and including two models that, respectively, fit the categorical and continuous versions of the number of children with serious conditions variable), and a final model identified as preferred by the model fit statistics.

## Results

**Figure 1.**
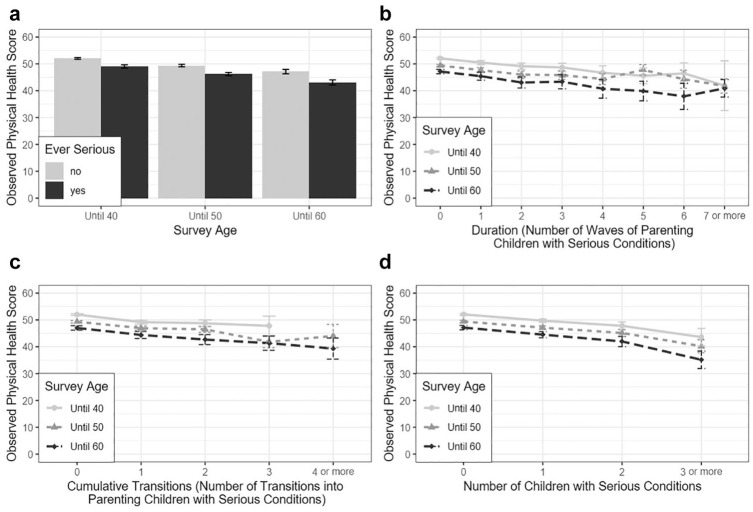
Mother’s Physical Health by Measures of the Intensity of Her Parenting Experience (National Longitudinal Survey of Youth: 1979, n = 8,305). (a) By “Ever Serious” Parenting Status. (b) By Duration. (c) By Cumulative Transitions. (d) By the Number of Children with Serious Conditions.

To begin, we offer a descriptive picture of the associations between mothers’ physical health and various measures of their experiences raising children with serious conditions (Figure 1). For clearer comparisons and interpretations, we separately report the results for each of the 40, 50, and 60 survey ages and combine larger values for duration (≥7 waves, accounting for <2%) and cumulative transitions (≥4 waves, accounting for <1%). Clear from the panels in Figure 1 is the expected health differences across age ranges. Calculations of mean differences showed that as mothers aged from 40 to 50 and from 50 to 60, their physical health scores declined by approximately 3 points during each interval. Our focus here is on patterns within each age category.

Consistent with prior research and across all age ranges, mothers who had experienced parenting a child with serious conditions reported lower physical health scores than those who had not (Figure 1, Panel A). Importantly, a mother’s physical health score decreased as duration, cumulative transitions, and number of children with serious conditions increased (Figure 1, Panels B–D). These declines were statistically significant for all three variables across the age ranges. They also were incremental and not driven by the difference between zero and positive values. In Figure 1, Panel D, for example, mothers who had two children with serious conditions had poorer physical health (across ages) than those who had three or more. Overall, Figure 1 provides preliminary evidence that intensity of the experience of raising children with serious conditions has health implications, not simply the experience itself.

### Examining Three Conceptualizations of Intensity of the Maternal Experience

To more rigorously test these preliminary patterns, we turned to linear fixed-effect regression models relevant to the first aim of testing the hypotheses about intensity of the maternal experience of raising children with serious conditions. Table 2 presents the LL, AIC, and BIC fit statistics for 12 model specifications, each based on different combinations of predictors related to mothering children with serious conditions. Most models interacted the ever serious variable with a selected intensity measure, following the internal moderation approach.

**Table 2.** Model Fit Statistics for Linear Fixed-Effect Models Predicting Mother’s Physical Health Score (National Longitudinal Survey of Youth: 1979, *n* = 8,305).

Model	Main Predictor(s)	*df*	LL	AIC	BIC
A	Ever	12	−25,928.4	51,880.8	51,965.1
B	Ever, ever × Duration	13	−25,916.6	51,859.2	51,950.5
C	Ever, ever × Cumulative transitions	13	−25,909.8	51,845.6	51,937.0
D	Ever, ever × Number of children with serious conditions (categorical)	14	−25,908.5	51,845.0	51,943.3
E	Ever, ever × Number of children with serious conditions	13	−25,909.3	51,844.6	51,935.9
F	Ever, ever × Duration, ever × Cumulative transitions	14	−25,909.7	51,847.4	51,945.7
G	Ever, ever × Duration, ever × Number of children with serious conditions (categorical)	15	−25,906.1	51,842.3	51,947.6
H	Ever, ever × Duration, ever × Number of children with serious conditions	14	−25,907.2	51,842.5	51,940.8
I	Ever, ever × Cumulative transitions, ever × Number of children with serious conditions (categorical)	15	−25,902.3	51,834.5	51,939.9
J	Ever, ever × Cumulative transitions, ever × Number of children with serious conditions	14	−25,903.5	51,834.9	51,933.3
K	Ever, ever × Duration, ever × Cumulative transitions, ever × Number of children with serious conditions (categorical)	16	−25,902.2	51,836.5	51,948.9
L	Ever, ever × Duration, ever × Cumulative transitions, ever × Number of children with serious conditions	15	−25,903.4	51,836.9	51,942.2

*Note:* All models adjust for covariates described in Table 1. LL = Log-likelihood; AIC = Akaike information criterion; BIC = Bayesian information criterion.

Three key findings emerged from the model selection process. First, improvements in LL, AIC, and BIC for Models B through E compared to Model A indicated that each conceptualization of the intensity of raising a child with a serious condition—specifically, duration, cumulative transitions, and the number of affected children—independently predicted lower maternal health scores above and beyond the simplified measure of whether a mother has ever had such an experience. Second, compared with duration (BIC = 51,950.5, AIC = 51,859.2), cumulative transitions (BIC = 51,937.0, AIC = 51,845.6) and the number of children with serious conditions (BIC = 51,935.9, AIC = 51,844.6) appeared to be particularly predictive of maternal physical health, as evidenced by more substantial reductions in BIC when these variables were added to the baseline model. Third, the BIC statistic suggested that Model J, which included cumulative transitions and the number of affected children (continuous) but not the duration measure, was the preferred model. The preference was further supported by the LL and AIC statistics, both of which remained largely unchanged with or without the duration measure (e.g., LL = −25,903.5 vs. −25,903.4; AIC = 51,834.9 vs. 51,836.9). Models I and K, which used a categorical measure of the number of children with serious conditions, yielded better fit according to their larger LL statistics. But we considered Model J the optimal choice due to its greater parsimony, as indicated by its smaller BIC statistic (51,933.3 vs. 51,939.9) and an essentially equal AIC statistic (51,834.9 vs. 51,834.5).

Based on this model-fitting exercise, Table 3 presents six selected models for closer examinations of their coefficient estimates. As a baseline, Model 1 replicated the negative association between maternal physical health and ever parenting children with serious condition reported in past studies. Conditional on covariates, Model 1 showed a nonsignificant (β = −.87, *p* < .1) coefficient for having parented at least one child with serious conditions. Compared with existing literature, the weaker significance level was unsurprising because the current model only considered the same mother’s changes in maternal status between ages 40 to 60, whereas many mothers (60% in our sample) had already experienced this particular maternal status before age 40. Indeed, results from random-effects models (Appendix A in the online version of the article), which mixed between- and within-person effects, suggested a stronger negative association for the ever serious variable (β = −2.01, *p* < .0001). These results illustrate the stringency of the fixed-effects approach.

**Table 3.** Results from Linear Fixed-Effect Models Predicting Mother’s Physical Health Score by Their Experience of Child’s Serious Conditions (National Longitudinal Survey of Youth: 1979, *n* = 8,305).

	Model 1		Model 2		Model3		Model 4		Model 5		Model 6	
	β	*SE*	β	*SE*	β	*SE*	β	*SE*	β	*SE*	β	*SE*
Raising child with serious condition												
Ever	−.87	.46	−.17	.50	.51	.55	−.79	.46	1.57*	.70	1.66*	.70
Ever × Duration			−.63***	.18								
Ever × Cumulative transitions					−1.43***	.32					−.94*	.37
Ever × Number of children with serious conditions categorical (reference = Ever × 1)												
Ever × 2							−1.86**	.63				
Ever × 3 or more							−5.63***	1.27				
Ever × Number children with serious conditions (continuous)									−2.32***	.50	−1.56**	.59
Socioeconomic circumstances												
Number of weeks working per year	.13***	.03	.13***	.03	.12***	.03	.13***	.03	.13***	.03	.13***	.03
Family income (IHS transformed)	.69*	.29	.66*	.29	.68*	.30	.64*	.29	.64*	.29	.64*	.29
Maternal covariates												
Number of children overall	1.63	.95	1.65	.95	1.69	.95	1.79	.95	1.79	.95	1.77	.95
Marital history (reference = stably married)												
Never married	.24	1.16	.25	1.16	.28	1.16	.20	1.16	.25	1.15	.27	1.15
Disrupted	.04	.54	.04	.54	.07	.54	.07	.54	.06	.54	.07	.54
Ever smoked	−1.59	1.09	−1.66	1.09	−1.68	1.09	−1.64	1.09	−1.58	1.09	−1.65	1.09
Ever in poverty	−.61	.65	−.68	.65	−.69	.65	−.63	.65	−.65	.65	−.69	.65
Years of education	.02	.21	.03	.21	.04	.21	.03	.21	.02	.21	.04	.21
Survey age (reference = 40)												
Age 50	−3.38***	.21	−3.16***	.22	−3.14***	.22	−3.24***	.22	−3.23***	.22	−3.12***	.22
Age 60	−6.46***	.31	−6.09***	.33	−5.96***	.33	−6.18***	.32	−6.16***	.32	−5.93***	.33
Constant	36.87***	4.81	37.26***	4.78	36.62***	4.78	37.02***	4.78	37.09***	4.80	36.86***	4.77
Square root of the variance: Level 2	8.31		8.22		8.26		8.23		8.23		8.22	
Square root of the variance: Level 1	7.37		7.36		7.35		7.35		7.35		7.35	
*n* at Level 2: mothers	3,682		3,682		3,682		3,682		3,682		3,682	
*n* at Level 1: occasions	8,305		8,305		8,305		8,305		8,305		8,305	
BIC	51,965		51,951		51,937		51,943		51,936		51,933	
AIC	51,881		51,859		51,846		51,845		51,844		51,835	

*Note:* AIC = Akaike information criterion; BIC = Bayesian information criterion; IHS = inverse hyperbolic sine.

* *p* < .05, **p < .01, ***p < .001.

Model 2 introduced an interaction between the ever serious status and the duration of parenting at least one child with serious conditions. To reiterate, this internal moderation strategy—interacting the variables without including the main effect of duration—tested the effect of extended duration among mothers in this role while using the full sample to estimate coefficients for other covariates. Model 2 revealed a strong negative association between this duration variable and maternal physical health. Given the biennial nature of the duration measure, the coefficient (β = −.63, *p* < .0001) implies that for a mother with a child with a serious condition, each additional year of such parenting was associated with a decline in her health score by .31 (= .63 / 2) after accounting for time-varying covariates and all time-invariant unobservable confounds. Furthermore, the original maternal status variable was no longer significant, suggesting that within-person variations in health were better captured by the duration measure than by this binary measure.

Model 3 focused on cumulative transitions, which had a negative and significant (β = −1.43, *p* < .001) coefficient. Controlling for time-varying covariates and unobserved time-invariant characteristics, each additional transition of a child from not having a serious condition to having a serious condition was associated with decreases in the maternal physical health score by 1.43 points. This association was larger in magnitude than the association for duration observed in Model 2. Additionally, a model including both duration and cumulative transitions (Appendix A, in the online version of the article) revealed that the duration coefficient was not significant (β = −.10, *p* = .26) and that the transition measure was (β = −1.30, *p* < .01). This result suggests that within-person, experiencing multiple “on” and “off” transitions in a child’s serious condition was more adversely associated with maternal health than the overall duration of this experience.

Models 4 and 5 focused on the potential health implications of having multiple children with serious conditions. These models used categorical and continuous measures, respectively, among mothers who ever had a child with a serious condition. Both models revealed statistically significant inverse associations between these focal variables and maternal health. Model 4 estimated that, controlling for time-varying and time-invariant variables, a mother who had two children with serious conditions reported a physical health score 1.86 points lower (*p* < .01) than when she had only one child with serious conditions. This difference was 5.63 points (*p* < .001) when she had three or more such children, larger than the corresponding association of a decade of aging (β = −3.24 for ages 40 vs. 50 and β = −2.94 for ages 50 vs. 60). Similarly, Model 5, which switched to a continuous measure, showed that each additional child with a serious condition in the family was associated with decreases in the mother’s predicted physical health by 2.32 points among mothers with at least one child with such a condition. The expected health difference between mothers of one child with serious conditions and those with none was .75 points (1.57–2.32 in Model 5), a disparity that was smaller in magnitude than the observed effects of having multiple children with serious conditions. These results are in line with expectations of intensity—that the link between having a child with a serious condition and mothers’ physical health would intensify as the number of children with serious conditions increased.

**Figure 2.**
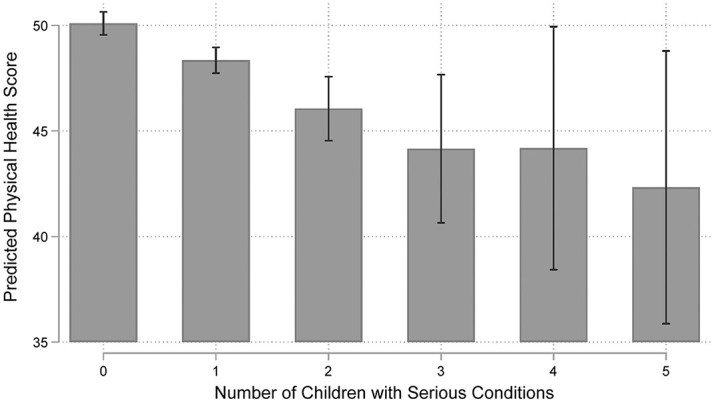
Expected Mothers’ Physical Health by the Number of Children with Serious Conditions (National Longitudinal Survey of Youth: 1979, n = 8,305). *Note*: Predicted values derived from Model 6.

As explained previously, the fit statistic indicated that Model 6 was the preferred model. It included both cumulative transitions and the number of children with serious conditions as a continuous predictor. In this preferred model, the significance level of each of the two focal variable was slightly attenuated from .001 to a .05 level, although both variables continued to have large and negative associations with maternal physical health (β = −.94, *p* < .05 for transitions; β = −1.56, *p* < .05 for the number of affected children). Among mothers who ever assumed this maternal role, each additional transition into having a child with a serious condition was associated with a .94 point decrease in mother’s physical health score. At the same time, each additional child with serious conditions was associated with a decrease in the mother’s physical health score of 1.56 points. Figure 2 shows the predicted values of the mother’s physical health as a function of the number of children with serious conditions. A given mother who did not have a child with a serious condition had a predicted health score of 50.09. Scores dropped to 48.34, 46.05, and 41.14 respectively as she had one, two, or three or more children with serious conditions. Although this decrease was gradual for mothers with one or two affected children, it became more pronounced for families with three or more.

Regarding covariates, employment status and family income tended to be most (positively) associated with physical health across models. The consistently negative coefficients for age across models reflected the anticipated declines in physical health due to aging. Finally, results on the variance structures confirmed the need to explicitly model within-person dependence of the data. All models had a residual intraclass correlation coefficient of over 50% (e.g., $\frac{8.22^{2}}{8.22^{2}+7.35^{2}}$ = 56%), meaning that within-person resemblance across time accounted for over half of the residual variations in the dependent variable.

### Exploring Variability of Maternal Intensity by Socioeconomic Circumstances

For the second aim, we elaborated the fixed-effects linear regression models to test whether the intensity of raising children with serious conditions would have weaker associations with maternal health in the context of higher incomes and labor force participation. For these modeling steps, we did not use the internal moderator approach to avoid the complexity of interpreting three-way interactions. Instead, we tested interactions between the potential socioeconomic moderators and each measure of intensity (i.e., duration, cumulative transitions, and the number of children with serious conditions in both categorical and continuous forms) in the subsample of mothers who had at least one child with serious conditions. For each dimension, we tested one interaction per model while controlling for the other two intensity variables. Doing so led to 10 models (5 × 2). Table 4 presents the five that show statistically significant results (*p* < .05). For the remaining models, see Appendix B in the online version of the article.

**Table 4.** Results from Linear Fixed-Effects Models for the Moderating Roles of Family Income and Labor Force Participation (National Longitudinal Survey of Youth: 1979, *n* = 3,344).

	Model 7		Model 8		Model 9		Model 10		Model 11	
	β	*SE*	β	*SE*	β	*SE*	β	*SE*	β	*SE*
Raising child with serious condition										
Duration	.08	.33	.08	.32	.10	.33	.09	.32	.05	.33
Cumulative transitions	−9.67***	2.87	−9.69***	2.87	−2.72**	.86	−2.70**	.86	−.79	.59
Number of children with serious conditions	−1.72*	.73			−1.58*		−1.23	.89	−4.63**	1.71
Number of children with serious conditions categorical (reference = 1)										
2			−1.21	.89			−1.23	.89	−4.63**	1.71
3 or more			−4.35**	1.68			−3.89*	1.68	−3.14	2.78
Socioeconomic circumstances										
Number of weeks working per year	.15**	.04	.15**	.04	.06	.05	.06	.05	.13**	.05
Family income (IHS transformed)	−.39	.69	−.40	.68	.86	.53	.85	.53	.94	.53
Number of children with serious conditions categorical (reference = 1)										
2			−1.21	.89			−1.23	.89	−4.63**	1.71
3 or more			−4.35**	1.68			−3.89*	1.68	−3.14	2.78
Interactions										
Cumulative transitions × Family income (IHS transformed)	.83**	.26	.86**	.27						
Cumulative transitions × Number of weeks working per year					.06**	.02	.06**	.02		
Number of children with serious conditions categorical × Number of weeks working per year (reference = 1 × Number of weeks working per year)										
2 × Number of weeks working per year									.11*	.04
3 or more × Number of weeks working per year									−.07	.09
Constant	49.07***	1.78	46.60***	1.74	38.66***	9.89	36.91***	9.90	34.60***	9.85
Square root of the variance: Level 2	9.57		9.57		9.52		9.52		9.61	
Square root of the variance: Level 1	8.00		8.00		8.00		8.01		8.01	
*n* at Level 2: mothers	1,708		1,708		1,708		1,708		1,708	
*n* at Level 1: occasions	3,344		3,344		3,344		3,344		3,344	
BIC	21,105		21,110		21,105		21,112		21,122	
AIC	21,013		21,013		21,013		21,014		21,018	

*Note:* The BICs and AICs for the baseline model without interactions are 21,117 and 21,032, respectively, if the number of children with serious conditions is modeled continuously, and 21,123 and 21,031, respectively, if it is modeled categorically. The sample is restricted to mothers who had parented a child with serious conditions by the time their health is measured (ever serious = 1). The models include all covariates fitted in Table 3, with unchanged significance levels as in Table 3. IHS = inverse hyperbolic sine; AIC = Akaike information criterion; BIC = Bayesian information criterion.

* *p* < .05, **p < .01, ***p < .001.

**Figure 3.**
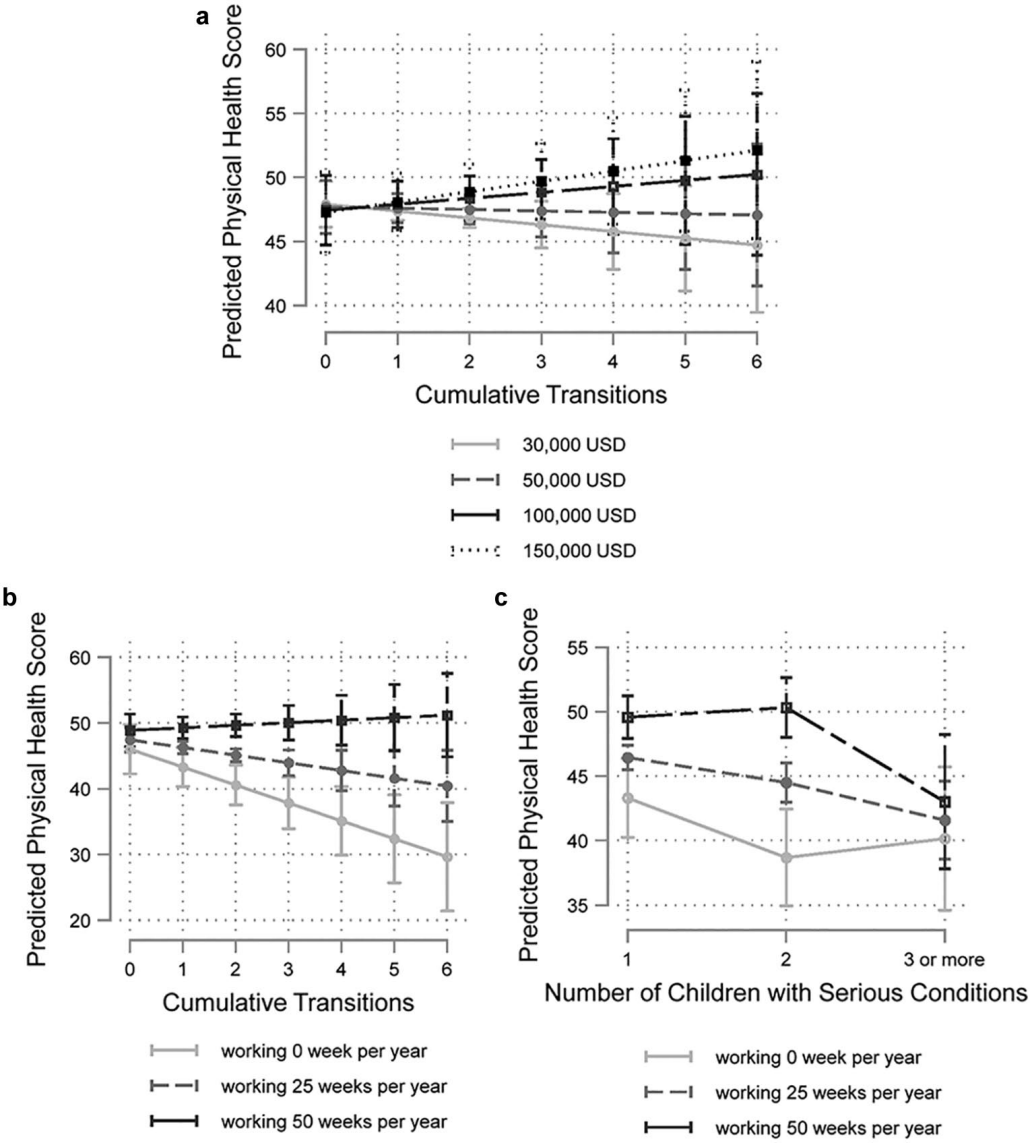
Interactions between Maternal Intensity and Socioeconomic Circumstances (National Longitudinal Survey of Youth: 1979, n = 3,344). (a) Interacting Cumulative Transitions with Family Income. (b) Interacting Cumulative Transitions with Labor Force Participation. (c) Interacting Number of Children with Serious Conditions with Labor Force Participation.

Beginning with family income, we found a significant interaction with cumulative transitions only. In Models 7 and 8, which, respectively, controlled for the continuous and categorical versions of the number of children with serious conditions, this interaction was positive and statistically significant (β = .86, *p* < .01 for both models). The association between having more cumulative transitions into this maternal role and poorer maternal health weakened as family income increased. To illustrate, Figure 3a presents predicted values of maternal health based on the interaction in Model 7.

Turning to labor force participation, Models 9 and 10 revealed positive and significant interactions between the number of transitions into having a child with a serious condition (whether categorical or continuous) and the number of working weeks per year. These interactions followed the same pattern as family income—the association between having more cumulative transitions into this maternal role and poorer maternal health weakened as employment increased. Figure 3b depicts this interaction graphically. If a mother worked full-time for approximately 50 weeks per year, her predicted health score did not change much as the number of transitions into raising a child with a serious condition increased. For a mother who did not work during a typical year (0 weeks per year), her physical health score dropped by 2.7 points (*p* < .01) with each additional transition into this maternal experience.

Model 11 captured the interactions between labor force participation and the number of children with serious conditions. Notably, although the continuous measure of the number of children with serious conditions did not significantly interact with labor force participation, the categorical measure did (*p* < .05), as indicated by a joint *F* test. This interaction was driven by the weaker association between having two children with serious conditions (compared to one) and maternal health when the number of weeks working per year was higher. This pattern did not extend to the comparison of having three or more children with serious conditions versus having one such child. Figure 3c depicts this significant contrast in which the potential buffering role of labor force participation in the health of mothers of children with serious conditions disappears after a certain level of intensity in the maternal experience is surpassed.

## Discussion

Prior research from a life course perspective has documented that the health implications of raising children with serious conditions reflect dosage effects that accumulate over time. Building on this foundation, our study sought to dissect these cumulative processes and explore contextual factors that conditioned their variability across populations. To achieve these goals, we developed three distinct conceptualizations of what we called the “intensity” of this mothering experience: the duration of serious conditions, the number of transitions into this maternal role, and the number of children with serious conditions. We then examined how the associations between these measures of intensity and maternal health varied across socioeconomic stratifiers, including family income and labor force participation.

Table 5 summarizes the results of this hypothesis testing about the role of intensity in health and its relation to socioeconomic functioning. In line with the temporal contextualization tenet of the life course perspective emphasizing the significance of exploring continuity and change in long-term trajectories, all three measures of intensity were associated with poorer maternal physical health in fixed-effects models. Moreover, each proved to be a better predictor than the simplified measure of whether a mother ever assumed the role of parenting a child with serious conditions. Of the three, cumulative transitions and the number of children with serious conditions emerged as particularly salient, the latter association more in line with a cumulative strain perspective than the alternate resource dilution hypothesis. In line with the social contextualization of the life course perspective emphasizing the variability of long-term trajectories across social structures and ecological settings, more labor force participation moderated the association with health of both cumulative transitions and having two (vs. one) children with serious conditions, and family income similarly moderated the association with health of cumulative transitions. This pattern of moderation echoes resource substitution (Ross and Mirowsky 2006) in that health-promoting resources mattered more in the face of risks. It did not extend, however, to mothers raising three (vs. one) children with serious conditions or to other combinations of intensity and socioeconomic resources. Together, these findings raise new questions about the “career” of mothering children with serious conditions for future research and policy design. We pull out three such questions here for further discussion.

**Table 5.** Summary of Main Results (National Longitudinal Survey of Youth: 1979).

	Main Effect (with Internal Moderator, *n* = 8,305)		Interaction with Family Income (*n* = 3,344)		Interaction with Weeks Working per Year (*n* = 3,344)	
Intensity Measure	Statistical Significance	Improved Model Fit	Statistical Significance	Improved Model Fit	Statistical Significance	Improved Model Fit
Duration	Yes	No	No	No	No	No
Cumulative transitions	Yes	Yes	Yes	Yes	Yes	Yes
Number of children with serious conditions (categorical)	Yes	No	No	No	Yes	Yes
Number of children with serious conditions	Yes	Yes	No	No	No	No

First, why did the number of transitions and number of children dimensions of the intensity of this maternal role outweigh the duration in this role? This difference in the temporal contextualization of life course pathways may reflect the underlying frame of instability versus equilibrium. Specifically, transitioning in and out of caregiving or adding new children to this long-term maternal pathway can be stressful and reduce the chances of reaching a “new normal.” Unlike a continuously diagnosed status, where strains accumulate gradually and care routines may stabilize, experiencing “on” and “off” switches and the addition of each affected child represent new disruptions that need to be coordinated. Alternatively, longer duration could allow for adaptation over time. In this scenario, the shocks could take the bigger toll on health rather than the more stable (even challenging) state, with each new shock adding to the strain evenly rather than marginally (as might be suggested by resource dilution and related models from the sibling literature).

This pattern echoes findings in family structure research. Changes in family structure tend to be more developmentally significant for children and mothers than structure itself. Even family structures commonly thought of as disadvantaged (e.g., single-mother households) can achieve a level of stability that supports adjustment and functioning than the more destabilizing force of moving from one family structure to another (Cavanagh and Fomby 2019). These insights suggest that support services for mothers raising children with serious conditions should focus on periods of transition or role expansion. To build on this evidence and its policy implications, future research needs to better account for key time-invariant variables (e.g., maternal or child’s age at first diagnosis) to clarify the balance between instability and equilibrium and capture trajectories of maternal health over long periods to reveal where such shocks have the most pronounced impacts.

Second, why did socioeconomic resources offer more buffering in the face of more transitions and additional children in this role than in the face of longer periods of this mothering experience? To use the language introduced previously, socioeconomic resources did more to moderate the shocks component of mothering children with serious conditions than the gradual accumulation component as a form of social contextualization of life course pathways. This specificity of such resource substitution patterns to the case of multiple shocks may be because socioeconomic resources help soothe disruptions and ease instability during transitions but do less to reduce strain once caregiving routines are established. In other words, socioeconomic resources could be especially helpful during times of heightened vulnerability, such as when a role is new or expanding. The logic of this resource substitution conceptualization from health research aligns with core themes of the more general theorization of social capital, which supposedly makes the biggest difference during times of uncertainty (Coleman 1988). Yet there appeared to be an upper limit on what support these resources could provide during such periods of heightened vulnerability, as seen in the lack of buffering for mothers with three or more children with serious conditions.

These patterns highlight a prime target for policy intervention—socioeconomically disadvantaged mothers of children with serious conditions during transitional periods. Interventions could involve the cultivation of socioeconomic resources as buffers or the identification of alternate support channels. To further this line of research, studies need to improve causal inference in assessing the main effects of dimensions of intensity and their interaction with socioeconomic resources, expand the conceptualization of resources to include psychosocial domains (e.g., supportive social networks) and institutional domains (e.g., access to services), break down generalized measures of maternal health into specific dimensions of adjustment and functioning that might be differentially reactive to this maternal experience, and refine child condition categorization to identify cases of heightened maternal vulnerability and potential for socioeconomic buffering.

Third, why was employment a more consistent moderator than income in the links between this maternal role and maternal health? In our view, labor force participation is a more multidimensional indicator of socioeconomic attainment than income, representing more than just financial resources. Although employment enhances economic security, it can also foster personal development (e.g., skills that facilitate coping and efficacy) and network building (e.g., social ties that increase information flow and support), among other things. Although working mothers of children with serious conditions may face greater work–family conflict, employment also allows them more resources to draw on to mitigate and cope with mothering strains (Prickett, Crosnoe, and Raley 2024). Thus, the buffering role of employment likely extends beyond economics, better capturing the resource substitution ideas of health promotion in the face of risk. Policy intervention could focus on cultivating the kinds of noneconomic buffering resources that paid employment can supply even among mothers outside the labor force, and future research should move beyond employment status to consider the conditions of employment that influence maternal well-being.

In conclusion, this study advances our understanding of how raising children with serious conditions shapes mothers’ physical health by demonstrating that the intensity of this experience matters (especially intensity in the form of shocks and not just in the form of duration) but operates differently across socioeconomic contexts (with lower health risks especially among mothers with higher income and paid employment in the face of shocks). These patterns underscore the value of fine-tuning conceptualizations of dosage when considering health risks in a population of interest and highlight the special case of mothers whose experience of raising children with serious conditions is intermittent or additive rather than stable, who are poorer, and who are outside the paid labor force.

## Supplemental Material

sj-docx-1-hsb-10.1177_00221465251353536 – Supplemental material for Life Course Dynamics in the Health of Mothers Raising Children with Serious Conditions Supplemental material, sj-docx-1-hsb-10.1177_00221465251353536 for Life Course Dynamics in the Health of Mothers Raising Children with Serious Conditions by Xuewen Yan and Robert Crosnoe in Journal of Health and Social Behavior
