# Supplementary material for: Life Course Dynamics in the Health of Mothers Raising Children with Serious Conditions
Source: J Health Soc Behav. 2025 Aug 3;67(2):253–71. doi: 10.1177/00221465251353536 (PMC13219777; doi:10.1177/00221465251353536)
Supplement: sj-docx-1-hsb-10.1177_00221465251353536 – Supplemental material for Life Course Dynamics in the Health of Mothers Raising Children with Serious Conditions [file sj-docx-1-hsb-10.1177_00221465251353536.docx]

**Journal** of **Health**

and **Social Behavior**

OFFICIAL JOURNAL OF THE AMERICAN SOCIOLOGICAL ASSOCIATION

**ONLINE SUPPLEMENT**

**to article in**

Journal of Health and Social Behavior

**Life Course Dynamics in the Health of Mothers Raising Children with Serious Conditions**

**Xuewen Yan**

*University of Texas, Austin*

**Robert Crosnoe**

*University of Texas, Austin*

Appendix A: Models predicting mental and physical health

|  | Model A1 | Model A2 | Model A3 |
| --- | --- | --- | --- |
|  | Mental health  (Fixed effect model) | Physical health  (Random effect model) | Physical health  (Fixed effect model) |
| Raising Child with Serious Condition |  |  |  |
| Ever | -0.22 (0.71) | -2.01^***^ (0.25) | 0.49 (0.55) |
| Ever × Duration | -0.10 (0.27) |  | -0.10 (0.26) |
| Ever × Number children with serious conditions | -0.072 (0.60) |  |  |
| Ever × Cumulative transitions | -0.47 (0.48) |  | -1.30^**^ (0.47) |
| Socioeconomic Circumstances |  |  |  |
| Number of weeks working per year | 0.053^*^ (0.025) | 0.17^***^ (0.01) | 0.13^***^ (0.03) |
| Log family income | 0.056 (0.30) | 0.33^*^ (0.14) | 0.74^*^ (0.31) |
| Maternal Covariates |  |  |  |
| Number of children overall | 1.17 (0.95) | 0.73^***^ (0.12) | 1.69 (0.95) |
| Marital history (Ref = stably married) |  |  |  |
| Never married | 0.60 (1.15) | -0.28 (0.47) | 0.28 (1.16) |
| Disrupted | -0.04 (0.54) | -0.93^***^ (0.28) | 0.074 (0.54) |
| Ever smoker | 0.30 (1.092) | -1.09^***^ (0.27) | -1.68 (1.09) |
| Ever in poverty | 0.51 (0.65) | -1.48^***^ (0.32) | -0.67 (0.65) |
| Years of education | 0.09 (0.21) | 0.29^***^ (0.06) | 0.04 (0.21) |
| Survey age (Ref = 40) |  |  |  |
| Age 50 | 0.046 (0.22) | -3.15^***^ (0.19) | -3.14^***^ (0.22) |
| Age 60 | 0.41 (0.33) | -5.96^***^ (0.26) | -5.97^***^ (0.33) |
| Constant | 45.35^***^ (4.78) | 38.99^***^ (1.53) | 36.51^***^ (4.78) |
| Sqrt. variance: Level 2 | 7.49 | 6.14 | 8.25 |
| Sqrt. variance: Level 1 | 7.35 | 7.37 | 7.35 |
| N at Level 2: Mothers | 3682 | 3682 | 3682 |
| N at Level 1: Occasions | 8305 | 8305 | 8305 |
| BIC | 51936.9 | 60262.3 | 51944.9 |

Standard errors in parentheses

^*^ *p* < 0.05, ^**^ *p* < 0.01, ^***^ *p* < 0.001

Appendix B: Linear fixed effect models for the moderating roles of family income and labor force participation (non-significant interactions)

|  | Model B1 | Model B2 | Model B3 | Model B4 | Model B5 |
| --- | --- | --- | --- | --- | --- |
|  | Physical health score | Physical health score | Physical health score | Physical health score | Physical health score |
| Raising Child with Serious Condition |  |  |  |  |  |
| Cumulative transitions | -0.90 (0.60) | -0.80 (0.60) | -0.80 (0.60) | -0.83 (0.59) | -0.79 (0.59) |
| Duration | -2.19 (1.37) | 0.09 (0.33) | 0.11 (0.33) | -0.51 (0.43) | 0.09 (0.33) |
| Number of children with serious conditions | -1.56^*^ (0.74) |  | -4.99 (4.06) | -1.56^*^ (0.74) | -2.44^*^ (1.17) |
| Number of children with serious conditions categorical (Ref = 1) |  |  |  |  |  |
| 2 |  | -9.31 (6.40) |  |  |  |
| 3 or more |  | -7.95 (9.74) |  |  |  |
| Socioeconomic Circumstances | |  |  |  |  |
| Log family income | 0.39 (0.69) | 0.81 (0.62) | 0.52 (0.86) | 0.95 (0.57) | 1.02 (0.57) |
| Number of weeks working per year | 0.14^**^ (0.04) | 0.14^**^ (0.04) | 0.14^**^ (0.04) | 0.09 (0.05) | 0.11 (0.06) |
| Interactions |  |  |  |  |  |
| Interactions |  |  |  |  |  |
| Duration × Log family income | 0.23 (0.14) |  |  |  |  |
| Number of children with serious conditions categorical × Log family income (Ref = 1 × Log family income) |  |  |  |  |  |
| 2 × Log family income |  | 0.82 (0.63) |  |  |  |
| (Continue from previous page) | |  |  |  |  |
| 3 or more × Log family income |  | 0.36 (0.99) |  |  |  |
| Number of children with serious conditions × Log family income |  |  | 0.34 (0.40) |  |  |
| Duration × Number of weeks working per year |  |  |  | 0.02+ (0.01) |  |
| Number of children with serious conditions × Number of weeks working per year |  |  |  |  | 0.027 (0.03) |
| Constant | 40.63^***^ (10.53) | 34.30^***^ (10.00) | 39.18^***^ (11.51) | 50.97^***^ (9.55) | 35.66^***^ (9.98) |
| Sqrt. variance: Level 2 | 9.53 | 9.52 | 9.53 | 9.54 | 9.55 |
| Sqrt. variance: Level 1 | 8.02 | 8.03 | 8.03 | 8.02 | 8.03 |
| N at Level 2: Mothers | 1708 | 1708 | 1708 | 1708 | 1708 |
| N at Level 1: Occasions | 3344 | 3344 | 3344 | 3344 | 3344 |
| Adjusted *R*^2^ | -0.735 | -0.738 | -0.738 | -0.735 | -0.738 |

Standard errors in parentheses

*+ p* < 0.1^*^ *p* < 0.05, ^**^ *p* < 0.01, ^***^ *p* < 0.001
